# A Modified DF2016 Criterion for the Fracture Modeling from Shear to Equibiaxial Tension

**DOI:** 10.3390/ma17040958

**Published:** 2024-02-19

**Authors:** Xiaona Xu, Ruqiang Yan, Xucheng Fang

**Affiliations:** School of Mechanical Engineering, Xi’an Jiao Tong University, 28 Xianning West Road, Xi’an 710049, China; xnxu18@xjtu.edu.cn (X.X.); xucheng.fang@stu.xjtu.edu.cn (X.F.)

**Keywords:** ductile fracture, DF2016 criterion, stress triaxiality, Lode parameter, advanced high-strength steel, sheet metal forming

## Abstract

This study introduces a modified DF2016 criterion to model a ductile fracture of sheet metals from shear to equibiaxial tension. The DF2016 criterion is modified so that a material constant is equal to the fracture strain at equibiaxial tension, which can be easily measured by the bulging experiments. To evaluate the performance of the modified DF2016 criterion, experiments are conducted for QP980 with five different specimens with stress states from shear to equibiaxial tension. The plasticity of the steel is characterized by the Swift–Voce hardening law and the pDrucker function, which is calibrated with the inverse engineering approach. A fracture strain is measured by the XTOP digital image correlation system for all the specimens, including the bulging test. The modified DF2016 criterion is also calibrated with the inverse engineering approach. The predicted force–stroke curves are compared with experimental results to evaluate the performance of the modified DF2016 criterion on the fracture prediction from shear to equibiaxial tension. The comparison shows that the modified DF2016 criterion can model the onset of the ductile fracture with high accuracy in wide stress states from shear to plane strain tension. Moreover, the calibration of the modified DF2016 criterion is comparatively easier than the original DF2016 criterion.

## 1. Introduction

With the continuous development of the aerospace and automotive industry, people are no longer satisfied with basic safety or strength requirements but hope that the materials can meet the requirements of weight reduction and energy conservation while meeting the strength standards. Advanced high-strength steel, aluminum alloys, and other metal materials have excellent material properties, especially in terms of strength and plasticity, making it possible to reduce weight, save energy, and meet safety standards. Therefore, they have shown excellent application prospects. However, a fracture that may occur during deformation processes, such as stamping and deep drawing, can pose a serious threat to the safety of practical applications. Therefore, it is necessary to study the deformation behavior of metals and to accurately predict the occurrence of fractures.

Researchers have developed many yield criteria to mathematically characterize the yield behavior of metals. First, many isotropic yield functions were developed to improve the modeling accuracy of yielding at different stress states of compression, shear, tension, etc., by considering the effect of pressure and the third stress invariant. These yield functions include the Tresca, von Mises, Drucker, Drucker–Prager, etc. For sheet metals, texture is formed during rolling, and sheet metals show dependence of plastic behavior on loading directions. Accordingly, many anisotropic yield functions were proposed. The Hill48 yield criterion [1] is one of the most representative research results, which accurately predicts the uniaxial and equibiaxial tensile strength along the rolling direction (RD), transverse direction (TD) and normal direction (ND) by introducing four anisotropic parameters based on the Huber–von Mises yield function. On this basis, the yield criteria after Hill48 increase the number of anisotropic parameters through linear transformation of stress tensors to improve the accuracy of the yield equation in characterizing plastic deformation. Barlat et al. [2] put forward the plane stress non-quadratic yield criterion to describe anisotropic metal sheets, such as aluminum alloy sheets. Barlat et al. [3,4,5] developed more accurate anisotropic yield functions based on a similar method to more accurately characterize the anisotropic behavior of metals and alloys. Other popular anisotropic yield functions were also proposed by Banabic et al. [6], Aretz and Barlat [7], Cazacu et al. [8], Cazacu [9], Yoshida et al. [10], Lou and Yoon [11], etc. Anisotropic hardening was extensively analyzed in the last 15 years by Stoughton and Yoon [12], Lee et al. [13], Park et al. [14], Hou et al. [15,16,17,18], Hu et al. [19,20,21,22,23], Du et al. [24], etc. Plastic behavior under various stress states was modeled recently by Hu et al. [25], Lou et al. [26,27], etc. These advances dramatically improve the plasticity modeling accuracy of metals under different loading directions and wide stress states.

Ductile fracture is increasingly investigated in the last 20 years since the 15 fracture experiments of AA2024-T351 by Bao and Wierzbicki [28]. Thereafter, many ductile fracture criteria were developed, including the modified Mohr–Coulomb criterion [29], the DF2012 [30], DF2016 [31], Mu [32], Ganjiani–Homayounfard [33], Hu–Chen [34], Zheng [35], Zhang [36], Quach [37], Shang et al. [38], etc. These criteria are expressed in a form of mixed stress and strain and based on micromechanisms of ductile fractures by nucleation, growth, and the coalescence of voids [39,40]. Stress-based ductile fracture criteria were proposed by Khan and Liu [41], Stoughton and Yoon [42], Mohr and Marcadet [43], sDF2016 [44], etc. An anisotropic ductile fracture was also studied in the last 10 years. Modeling approaches of anisotropic ductile fracture were proposed by Beese et al. [45], Luo et al. [46], Jia and Bai [47], and Lou and Yoon [48]. Park et al. [49] numerically studied ductile fracture modeling in pre-cracked tensile tests of SUS304L stainless steel. Baral et al. [50] modelled plasticity and ductile fracture of an Al-Si-Mg die-cast alloy. Bidadi et al. [51] investigated the effects of model mixity and the loading rate on the fracture behavior of cracked thin-walled 304L stainless steel sheets with large non-linear plastic deformations. Khan and Liu [52] proposed a ductile fracture criterion to consider strain rate and temperature effect. Wcislik and Lipies [53] reviewed the numerical modeling of void development in metals to investigate the mechanism of a ductile fracture during plastic deformation. Baral and Korkolis [54] investigated ductile fracture under proportional and non-proportional multiaxial loading. Alrasheedi et al. [55] investigated the tensile deformation and fracture of unreinforced AZ91 and reinforced AZ91-C at temperatures up to 300 °C. Ha et al. [56] characterized the ductile fracture of an aluminum sheet under proportional loading. Egidio et al. [57] analyzed the influence of microstructure on fracture mechanisms of the heat-treated AlSi10Mg alloy produced by laser-based powder bed fusion. Korkolis and Kyriakides [58] investigated the effect of the strain path on the failure of inflated aluminum tubes. Torabi et al. [59] investigated the fracture behavior of AA7075-AA6061 and AA7075-Cu friction-stir welded joint. Roth and Mohr [60] characterized the effect of the strain rate on the fracture of advanced high-strength steel.

In this study, the DF2016 fracture criterion is modified to model the fracture behavior of advanced metals from shear to equibiaxial tension. In the modified DF2016 criterion, the material constant *C*_3_ is equal to the fracture strain at equibiaxial tension, which can be easily measured by the bulging experiments. Therefore, the material calibration of the modified DF2016 criterion is relatively simple. The modified DF2016 criterion is applied to model the fracture behavior of an advanced high-strength steel of QP980. Five different specimens are tested to characterize plasticity and fracture behaviors from shear to equibiaxial tension with the strain measurement by the XTOP digital image correlation system. Plasticity is characterized by the Swift–Voce hardening law and the pDrucker function. The modified DF2016 criterion is calibrated with an inverse engineering approach. The predicted load–stroke curves with fractures are compared with the experimental results to evaluate the performance of the modified DF2016 criterion from shear to equibiaxial tension.

## 2. A Modified DF2016 Fracture Criterion

The DF2016 criterion is proposed to characterize fracture onset for sheet metals from shear to plane strain tension. It is expressed as below:

(1) $${\left(\frac{2\tau_{max}}{{\bar{\sigma }}_{VM}}\right)}^{C_{1}}{\left(\langle \frac{f\left(\eta ,L,C\right)}{f\left(1/3,-1,C\right)}\rangle \right)}^{C_{2}}{\bar{\varepsilon }}_{f}^{p}=C_{3}\;\;\;\langle x\rangle =\left\{\begin{array}{ll} x & if\;x\geq 0\\ 0 & if\;x<0 \end{array}\right.$$

with

(2) $$f\left(\eta ,L,C\right)=\eta +C_{4}\frac{\left(3-L\right)}{3\sqrt{L^{2}+3}}+C$$

where η is the stress triaxiality, L is the Lode parameter, $\tau_{max}$ is the maximum shear stress, ${\bar{\sigma }}_{VM}$ is the von Mises equivalent strain, and ${\bar{\varepsilon }}_{f}^{p}$ is the equivalent plastic strain at fracture. There are five fracture parameters of $C_{1}$, $C_{2}$, $C_{3}$, $C_{4}$, and C. The DF2016 criterion reduces to the DF2014 criterion when $C_{4}=1$ and the DF2012 criterion by setting $C_{4}=0$ and C=1/3. The DF2016 criterion can be reformulated in a form of the Lode parameter and stress triaxiality because the maximum shear stress normalized by the von Mises effective stress is solely a function of the Lode parameter as below:

(3) $${\left(\frac{2}{\sqrt{L^{2}+3}}\right)}^{C_{1}}{\left(\langle \frac{f\left(\eta ,L,C\right)}{f\left(1/3,-1,C\right)}\rangle \right)}^{C_{2}}{\bar{\varepsilon }}_{f}^{p}=C_{3}$$

In the DF2016 criterion, the material constant $C_{3}$ is equal to the equivalent plastic strain to fracture at uniaxial tension. The fracture strain at equibiaxial tension can be easily predicted by the bulging test with DIC technique. Therefore, the DF2016 criterion is modified so that $C_{3}$ is equal to the equivalent plastic strain at equibiaxial tension by bulging tests as below:

(4) $${\left(\frac{2}{\sqrt{L^{2}+3}}\right)}^{C_{1}}{\left(\langle \frac{f\left(\eta ,L,C\right)}{f\left(2/3,1,C\right)}\rangle \right)}^{C_{2}}{\bar{\varepsilon }}_{f}^{p}=C_{3}$$

In the numerical application of the ductile fracture criterion above, the von Mises equivalent stress and strain are computed again based on the stress components updated based on the yield function, which is used to describe the plastic deformation of metals. After that, the stress triaxiality and Lode parameter are then computed according to their definitions based on the von Mises equivalent stress to compute damage and fractures during plastic deformation.

## 3. Materials and Experiments

**Figure 1:**
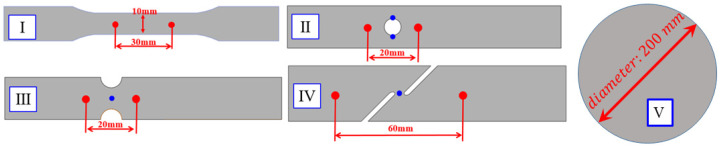
Four types of specimens [61]: (**I**) dogbone specimens; (**II**) specimens with central hole; (**III**) notched specimens; (**IV**) in-plane shear specimens; and (**V**) bulging specimens.

This part aims to collect mechanical behavior data under uniaxial tension, hole tension, plane strain tension, shear tension, and equibiaxial tension to assess the plastic behaviors of the QP980 steel. The material is manufactured by BAOSTEEL in Shanghai, China. The chemical composition of QP980 in weight percent is 0.2% C, 1.49% Si, 1.82% Mn, 0.011% P, 0.0043% S and 0.046% Al. The thickness of the steel was 1.0 mm. Five specimens were cut as shown in Figure 1 to characterize the mechanical properties of QP980 steels. These five specimens included the dogbone specimens, the specimens with a central hole, the notched specimens, the in-plane shear specimens, and the circular specimens. The first four specimens were tested with a universal material testing machine, and the deformation was measured with the XTOP DIC method. The circular specimens were used to conduct the bulging test. The dogbone specimens were used to characterize plasticity at uniaxial tension along three different loading directions. The specimens with a central hole were used to characterize fracture behavior under uniaxial tension, the notched specimens were used to characterize the fracture behavior under plane strain tension, the in-plane shear specimens were used to characterize fracture strain under shear, and the circular specimens for bulging tests were used to measure the fracture strain of the steel under equibiaxial tension. The dimensional information of the specimens was designed as shown in Figure 1, including the initial gauge length.

**Figure 2:**
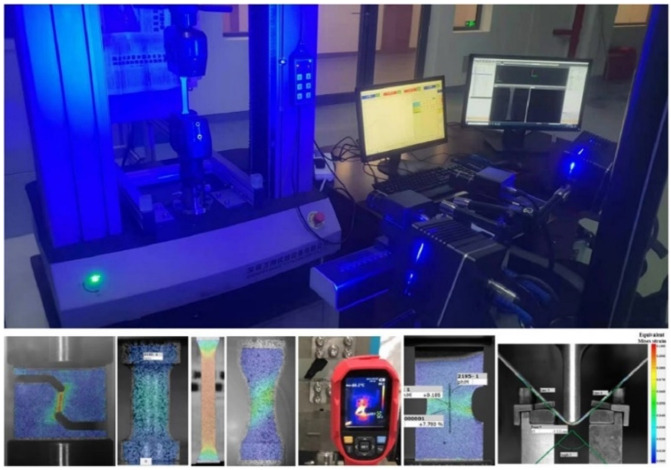
Universal mechanical testing system and the XTOP digital image correlation [62].

**Figure 3:**
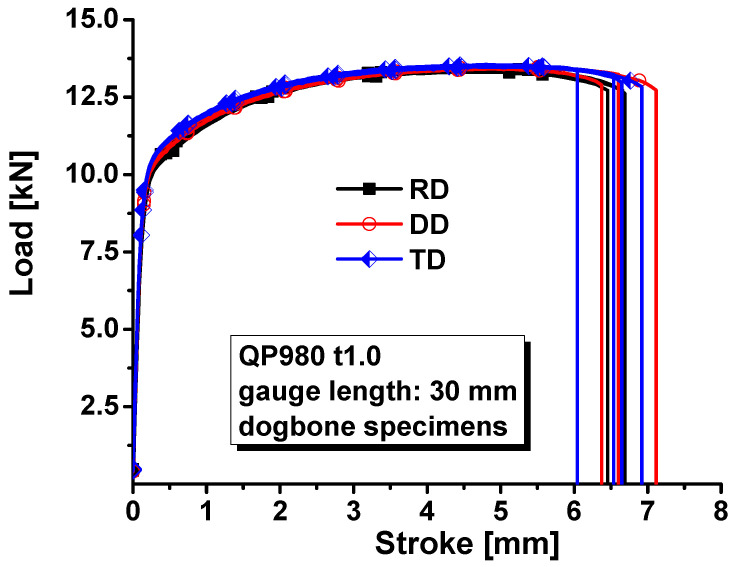
Load–stroke curves of QP980 for dogbone specimens.

**Figure 4:**
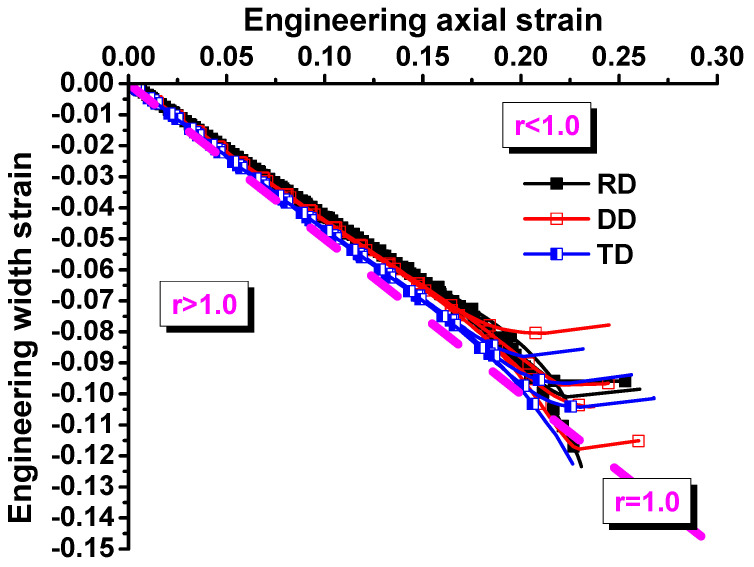
Relations between axial and width strain evolution of QP980 for dogbone specimens.

The universal mechanical testing system in Figure 2 was used to load the first four specimens. The loading velocity was 3.6 mm/min for the dogbone specimens to ensure that the strain rate during the tests was about 0.001/s. Deformation processes during experiments were recorded using the XTOP digital image correlation. Force during experiments was measured with a load cell. The measured force–stroke curves for the dogbone specimens were compared in Figure 3 along three directions of RD, DD, and TD. The evolution of plastic strain along the longitudinal and width directions is shown in Figure 4 to evaluate the anisotropic plastic deformation along the three directions. The comparison shows that the anisotropy in strength and plastic deformation is negligible. Therefore, the material was assumed to be isotropic in this study.

**Figure 5:**
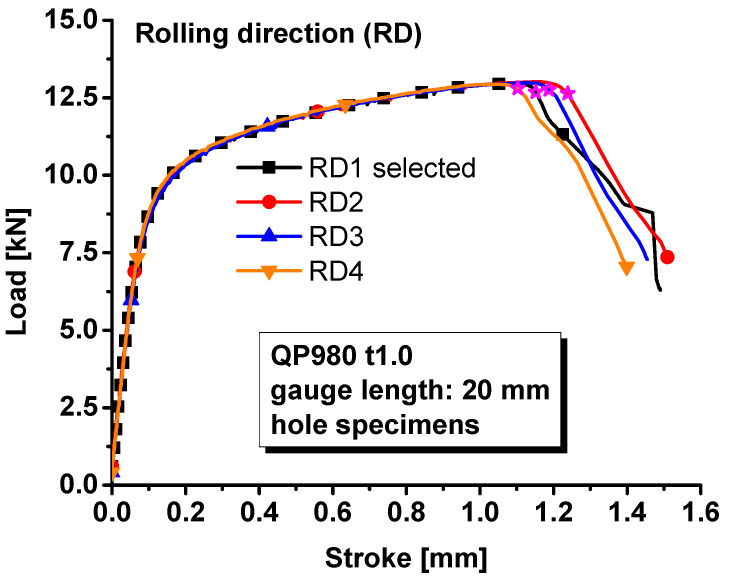
Load–stroke curves of QP980 for hole specimens.

**Figure 6:**
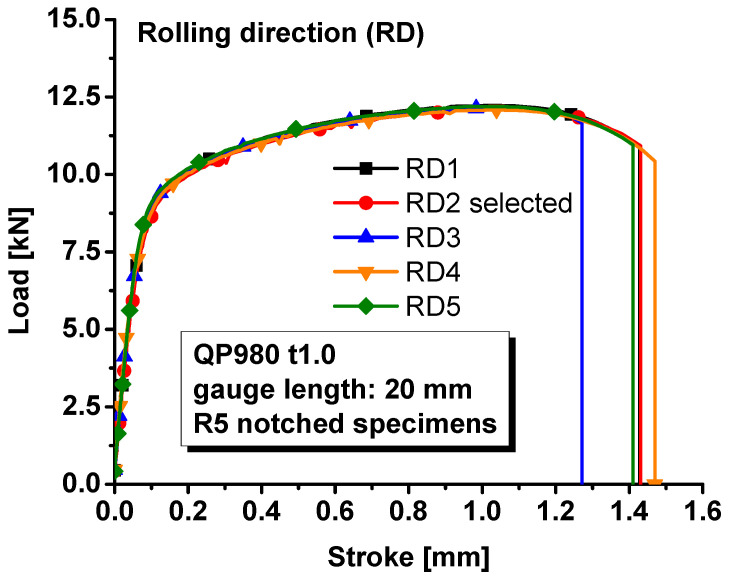
Load–stroke curves of QP980 for notched R5 specimens.

**Figure 7:**
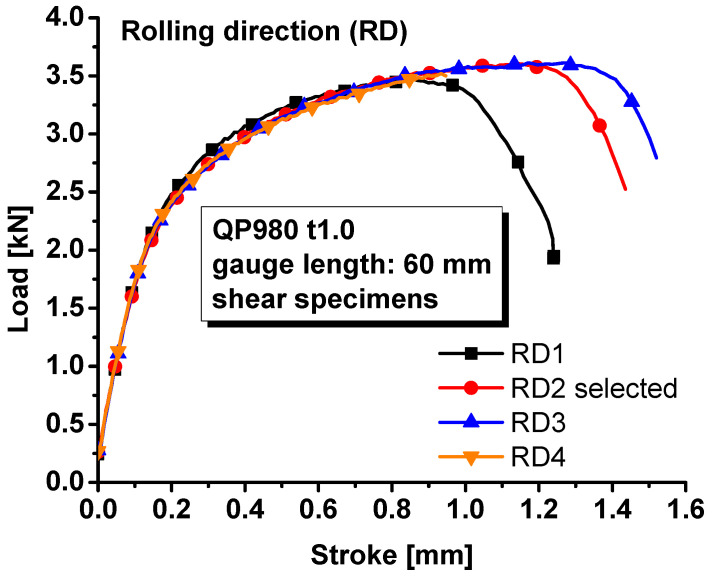
Load–stroke curves of QP980 for shear specimens.

The load–stroke curves were also measured for the specimens with a central hole, notched specimens, and shear specimens along the rolling direction since the material was assumed to be isotropic. The loading velocity was set as 0.5 mm/min for the specimens with a central hole, notched specimens, and the in-plane shear specimens so that the strain rate during the tests were about 0.001/s. The measured load–stroke curves were compared in Figure 5 for the specimens with a central hole, Figure 6 for notched specimens, and Figure 7 for the shear specimens. It was obvious that the repeatability of the tests was reliable regarding the hardening behavior of the material. However, the stroke at failure was not as repeatable as the hardening behavior, especially for the shear test. The poor repeatability in the stroke at failure may be due to manufacturing error, inhomogeneous microstructure, etc. In this study, the most repeatable experiments with mean stroke at failure were selected to represent the experimental results for different specimens. Therefore, test #1 was selected for the further analysis of the specimen with central hole and #2 for the notched and shear specimens.

**Figure 8:**
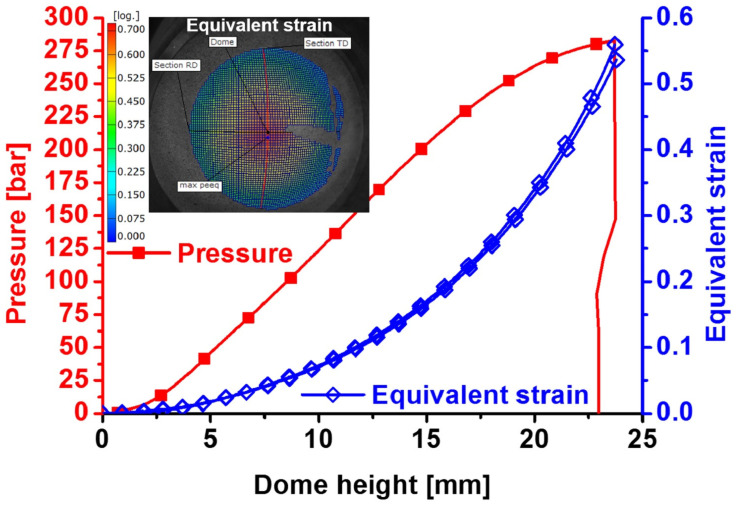
Bulging experimental results of QP980.

Bulging tests were conducted for the QP980 steel with the specimen V in Figure 1. The punch velocity was 5 mm/min. Three bulging tests were carried out to ensure the repeatability of the experiments. The pressure and dome height are plotted in Figure 8. The evolution of equivalent strain is also shown against the dome height in the figure. It was measured that the fracture strain at the dome was about 0.5361 for QP980. There were two fracture strains shown in the figure, and the smaller one was selected so that fracture prediction was somewhat conservative. All the strains from DIC were the von Mises equivalent strain. To be consistent, the von Mises equivalent strain and its increment were computed by the plastic strain increments to compute the damage and fracture during simulation of plastic deformation. According to the modified DF2016 criterion, this fracture strain was equal to the parameter *C*_3_ in the modified DF2016 criterion in Equation (4).

## 4. Plasticity Modeling

**Figure 9:**
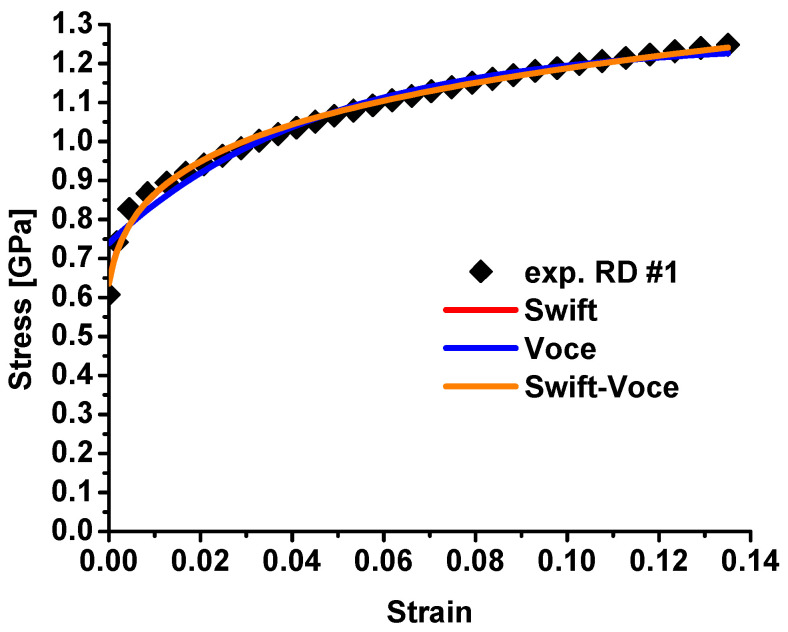
Comparison of the fitted hardening laws with experimental results for QP980.

The strain hardening behavior is characterized by the dogbone specimens and fitted with the Swift–Voce hardening law below in Equation (5). The fitted parameters are summarized in Table 1 for the Swift, Voce, and Swift–Voce hardening laws. The fitted hardening laws are used to predict the strain hardening of QP980 and compared with experimental results, as shown in Figure 9. In the finite element analysis, small elements with an edge size of about 0.1 mm are adopted for the severe plastic zones of these specimens. All the simulations are conducted with C3D8R brick elements. All the other settings in the simulation are identical with the experimental conditions for all three specimens. The Swift and Swift–Voce hardening law are almost overlapped. The Voce model predicts the worst flow curve for QP980. The comparison demonstrates that the Swift–Voce hardening law fits the experimental result with the best agreement compared to the Swift and Voce hardening laws.

(5) $$\bar{\sigma }=\alpha K{\left(e_{0}+{\bar{\varepsilon }}^{p}\right)}^{n}+\left(1-\alpha \right)\left(A-(A-B)exp\left(-C{\bar{\varepsilon }}^{p}\right)\right)$$

**Figure 10:**
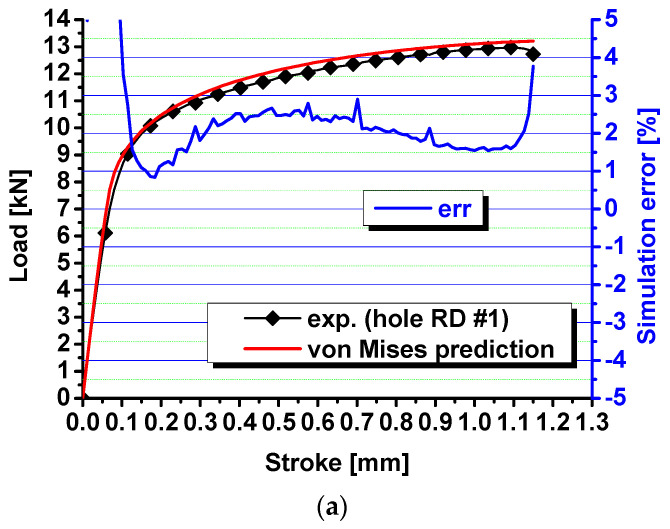

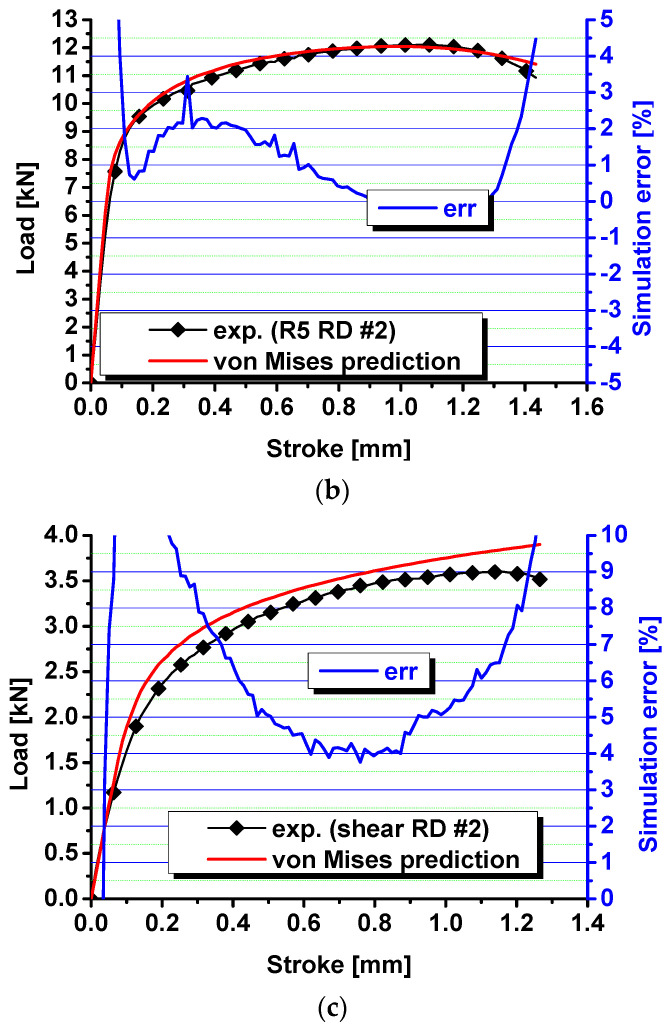
Comparison of the predicted load–stroke curves via von Mises function with experimental results for (**a**) specimens with a central hole; (**b**) notched specimens; and (**c**) shear specimens.

Then the fitted Swift–Voce law is used to predict the load–stroke responses of specimens with a central hole, notched R5 specimens, and the in-plane shear specimens. The numerical simulation is conducted with Abaqus/Explicit 6.14. The minimum element size is about 0.5 mm. The predicted results are compared with experimental results in Figure 10. The prediction errors by numerical simulation are also computed with respect to stroke increase. It is observed that the predicted force is about 2% larger than the experimental results for the specimens with a central hole, 1% higher than the experimental results for the notched R5 specimens, and 4%~9% higher than the in-plane shear specimen results. The simulation error is too big, especially for the in-plane shear specimens, and not acceptable. The big error is due to the fact that the von Mises yield function cannot take the effect of the stress state into account on yielding and plastic deformation. The Lode parameter is 0.0 for shear and plane strain tension, but the strain hardening behavior is characterized by uniaxial tension of dogbone specimens whose Lode parameter is −1.

The big load–stroke prediction error is due to the fact that the effect of stress states is not considered for the strength modeling from shear to plane strain tension. Therefore, the pDrucker yield function [26] is applied for QP980 to consider the effect of stress states on strength and expressed as follows:

(6) $$\bar{\sigma }\left(\sigma_{ij}\right)=a\left(bI_{1}+{\left(J_{2}^{3}-cJ_{3}^{2}\right)}^{1/6}\right)$$

with

(7) $$a=\frac{1}{b+\frac{1}{3}{\left(27-4c\right)}^{1/6}}$$

where $I_{1}$, $J_{2}$, and $J_{3}$ are the three stress invariants and a, b, and *c* are material parameters to adjust the yield surface. The computation of the parameter *a* in Equation (7) is based on the assumption that the strain hardening is characterized by uniaxial tensile tests by dogbone specimens. The *a* parameter can also be computed in a different form if the strain hardening curve is characterized at equibiaxial tension by the bulging tests. Details are suggested in the publication [26]. In the pDrucker function, there are three parameters to model the effect of stress states on the strength of QP980 steel sheets from shear to plane strain tension. These three parameters are calibrated with the flow curves under shear, uniaxial tension, and equibiaxial tension. By introducing the pressure effect and the dependence of the third stress invariant, the pDrucker function can predict different yield stresses in shear, uniaxial tension, and plane strain tension. This difference cannot be modeled by the von Mises yield function because the von Mises function only considers the effect of the second stress invariant on yielding.

**Figure 11:**
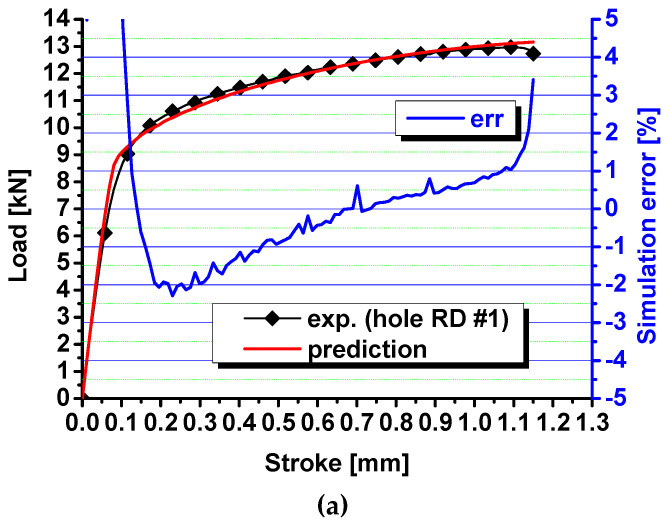

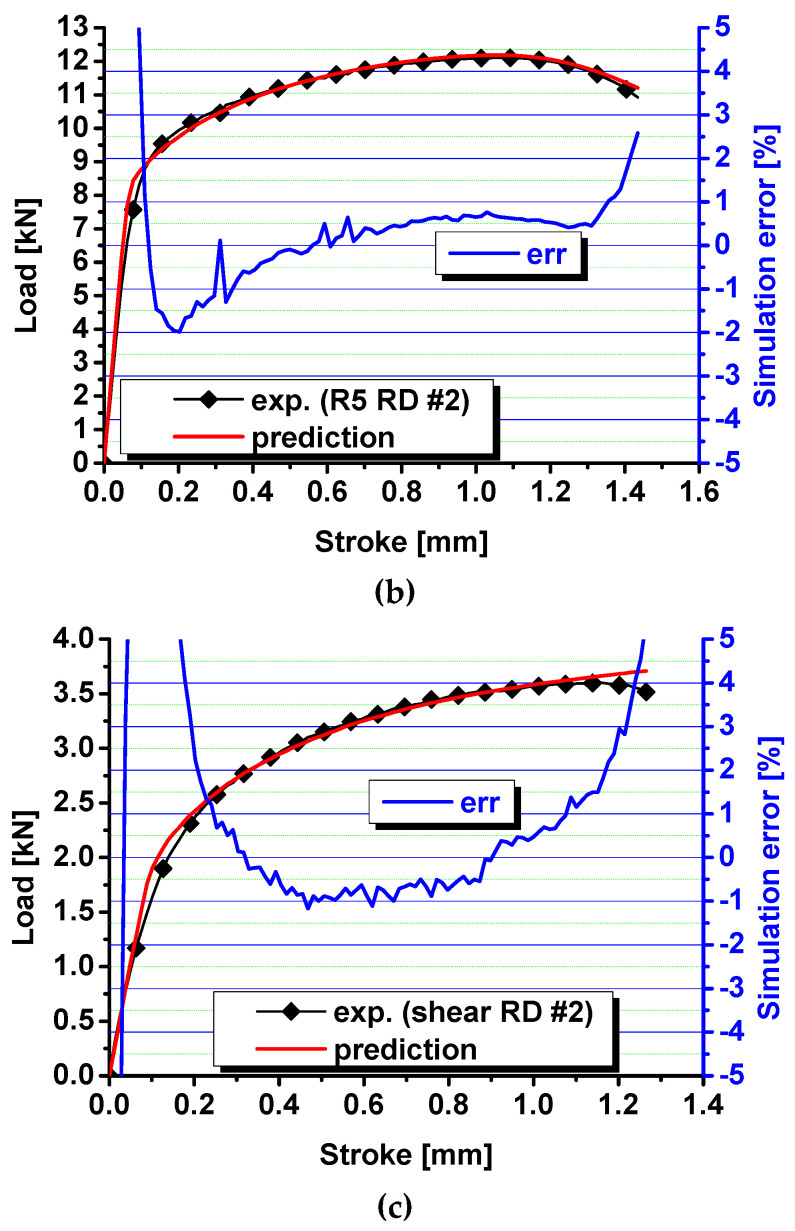
Comparison of the predicted load–stroke curves via the pDrucker function with experimental results for (**a**) specimens with a central hole; (**b**) notched specimens; and (**c**) shear specimens.

**Figure 12:**
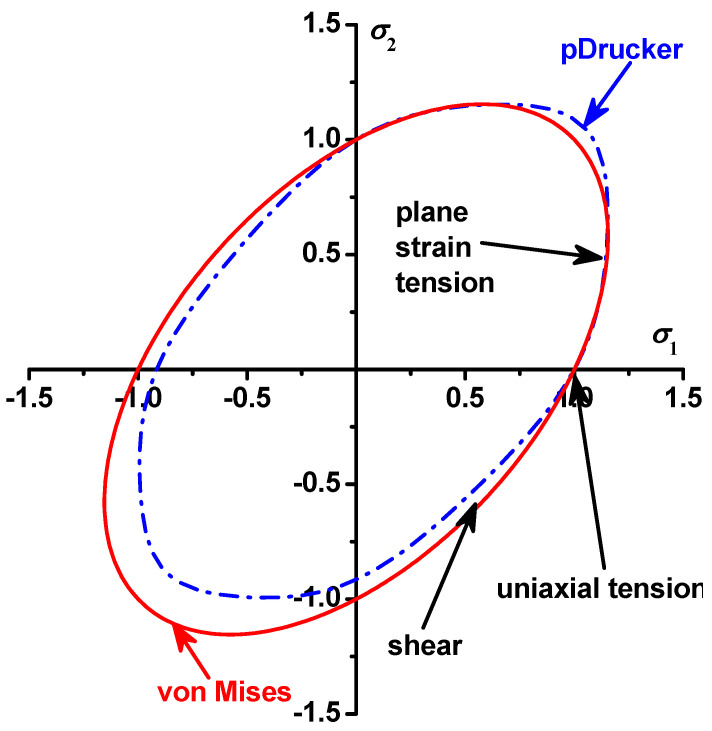
Comparison of the von Mises and pDrucker yield surfaces.

The pDrucker function and the Swift–Voce hardening law are calibrated with the inverse engineering approach [26] with the calibrated parameters in Table 2. Then, the load–stroke curves are predicted by the Abaqus/Explicit and compared with the experimental results in Figure 11. The prediction error is also computed and compared in the figure. The comparison shows that the error ranges from about −2% to 2% for the specimens with a central hole, from −1% to 1% for the notched specimens, and from −1% to 3% for the shear specimens. Compared with the prediction with the von Mises yield function, the prediction error for the load–stroke curves is significantly reduced by the pDrucker function, especially for the in-plane shear specimens, for which the error is reduced from 4%~9% to −1%~3%. Therefore, the Swift–Voce hardening law and the pDrucker function calibrated in Table 2 are used to model the plastic behavior of QP980 for these three specimens with stress states from the shear to plane strain tension. The significant reduction in the prediction error of the load–stroke curves is because the pDrucker yield function adjusts the relative strength of shear, uniaxial tension, and plane strain tension by optimizing the parameters of *a*, *b*, and *c* during inverse engineering approach, as shown in Figure 12, for the comparison of the von Mises and pDrucker yield surfaces. The error in the force–stroke curve for shear specimens is significantly reduced by the pDrucker yield function because the difference between the von Mises and pDrucker yield surfaces is very apparent, as shown in Figure 12. The yield surface difference under uniaxial and plane strain tension is not as obvious as that around shear. Therefore, the prediction accuracy improvement in the force–stroke curves is not obvious for specimens with a central hole and notched specimens. The error even increases slightly for the specimens with a central hole, which is due to the fact that the inverse engineering approach minimizes the total error in the load–stroke prediction for the three specimens. In the studied case, the total error is reduced, but the method sacrifices the prediction accuracy for the specimens with a central hole.

The hardening law was fitted for low strain values before necking based on the dogbone specimens, and the fitted strain hardening parameters are summarized in Table 1. However, the fitted Swift–Voce hardening law cannot accurately predict the reaction forces for shear specimens, specimens with a central hole, and notched specimens, as shown in Figure 10. The force–stroke curves predicted by the flow curve calibrated with the inverse engineering approach matches with the experimental results with higher accuracy, as shown in Figure 11. To further improve the prediction accuracy of the force–stroke curves for different specimens, the evolution of yield surfaces is suggested to be considered during plastic deformation at different stress states.

## 5. Fracture Modeling with the Modified DF2016 Criterion

The modified DF2016 criterion in Equation (4) is used to model the fracture behavior of QP980 from shear to equibiaxial tension. The material constant *C*_3_ is equal to the fracture strain at equibiaxial tension, which is measured with the bulging test to be 0.5361 with the help of the XTOP digital image correlation method. The other fracture parameters in the modified DF2016 criterion are calibrated with the inverse engineering approach and summarized in Table 3. The inverse calibration of fracture parameters is conducted by minimizing the error between the predicted fracture stroke and experimental results for shear, specimens with a central hole, and plane strain tension of notched specimens. The fracture stroke during the simulation is determined by the sharp drop of load–stroke curves during simulation.

In the implementation of the modified DF2016 criterion to the finite element simulation, the pDrucker yield function is used to describe the plastic deformation of the metal. The corresponding pDrucker equivalent strain is computed based on the pDrucker yield function to describe the strain hardening of the metal. The plastic strain increment components are obtained at the end of each integration and then used to compute the von Mises equivalent strain increment. The computed von Mises equivalent strain is used to compute damage and fractures based on the modified DF2016 criterion. All the equivalent strain used in damage and fracture computation is the von Mises equivalent strain because the calibration of the fracture criterion is based on the von Mises strain computed by DIC in different experiments.

**Figure 13:**
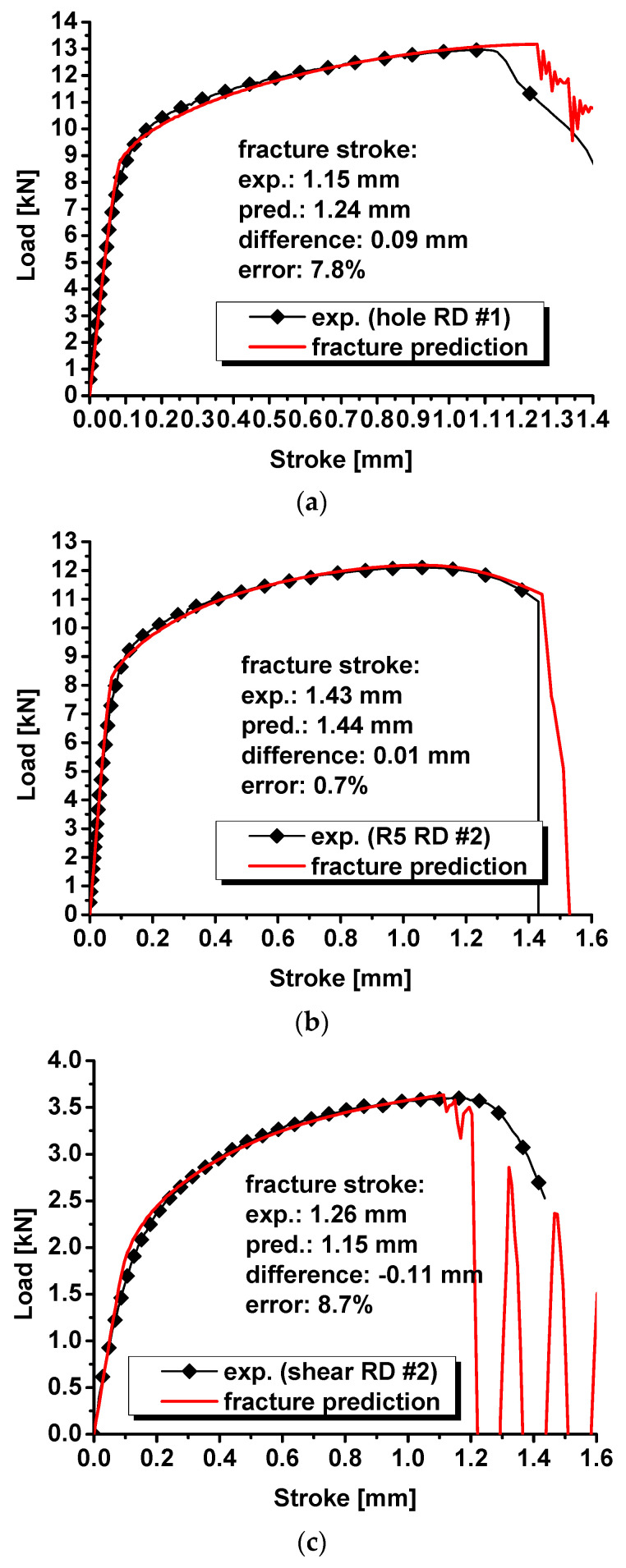
Comparison of the predicted load–stroke curves with the onset of ductile fracture via the modified DF2016 criterion with experimental results for (**a**) specimens with a central hole; (**b**) notched specimens; and (**c**) shear specimens.

The modified DF2016 criterion is implemented into ABAQUS/Explicit to predict the onset of the fracture for QP980 under various stress states from shear to plane strain tension. The load–stroke curves with an element deletion from the modified DF2016 criterion are predicted and compared with the experimental results of the specimens with a central hole, notched specimens, and shear specimens in Figure 13. For the specimens with a central hole, the predicted fracture stroke is 1.24 mm, while the experimental result is 1.15 mm. The difference between experiments and prediction is 0.09 mm, and the error is about 7.8% for the specimens with a central hole. For the notched specimens, the numerical prediction of the fracture stroke is 1.44 mm, and the experimental results is 1.43. The predicted fracture stroke is 0.01 mm longer than the experimental results, and the error is 0.7%. For the shear specimens, the predicted fracture stroke is 1.15 mm, while the experimental result is 1.26 mm. The experimental result is 0.09 mm higher than the prediction, and the error is 8.7%. Based on the comparison between prediction and experimental results in Figure 13, the prediction of the fracture stroke is all less than 10%. Considering the difficulty of the fracture prediction under complicated stress states, the prediction accuracy of less than 10% is definitely acceptable for engineering applications.

In addition, oscillation behaviors are observed for the force evolution in the simulation for specimens with a central hole, notched specimens, and shear. This is because all the numerical simulations are conducted with explicit formulation via ABAQUS/Explicit. The oscillation can be reduced by decreasing the mass scaling factor during the simulation or removed by the simulation with an implicit scheme via ABAQUS/Standard. However, simulations with ABAQUS/Standard cannot remove elements after a fracture.

The purpose of this study is to introduce the modified DF2016 criterion, which is relatively simple in parameter calibration compared to the original DF2016 criterion. Fracture prediction is not conducted in this study with the von Mises yield function because a simulation with the von Mises yield function results in a big error in the force–stroke curve prediction for the shear specimens. However, a fracture can be predicted with the modified DF2016 criterion with the von Mises yield function, but the fracture parameters of the modified DF2016 criterion need to be calibrated again to obtain good fracture prediction results. The key problem is that the predicted reaction force with the von Mises function is not as accurate as that with the pDrucker function.

## 6. Conclusions

This study proposed a modified DF2016 criterion to model a ductile fracture from shear to equibiaxial tension for sheet metals. The modified DF2016 criterion is applied to describe the onset of a fracture for four specimens of QP980 steel. The result shows that the modified DF2016 criterion predicts the ductile fracture with acceptable accuracy. In addition, the modified DF2016 criterion is user-friendly since the fracture parameter *C*_3_ is equal to the fracture strain at equibiaxial tension, which can be measured directly with the bulging test. According to the high accuracy and user-friendliness, the modified DF2016 criterion is suggested to be applied to model the fracture behavior of sheet metals from shear to equibiaxial tension.

## Figures and Tables

**Table 1:** Coefficients of Swift–Voce function.

	K [GPa]	e_0_	n	A [GPa]	B [GPa]	C	α
Swift	1.6562	0.0014	0.1451	\	\	\	\
Voce	\	\	\	1.2543	0.7393	21.5435	\
Swift–Voce	1.6562	0.0014	0.1451	1.2543	0.7393	21.5435	1.0262

**Table 2:** Coefficients of the Swift–Voce function and the pDrucker function calibrated with the inverse engineering approach.

pDrucker			Swift–Voce Hardening Law						
*a*	*b*	*c*	K [GPa]	e_0_	n	A [GPa]	B [GPa]	C	α
1.8769	−0.02486	1.2692	1.796	0.0080	0.1862	1.4050	0.6993	15.2893	0.5

**Table 3:** Fracture parameters of the modified DF2016 criterion calibrated with the inverse engineering approach.

*C*_1_	*C*_2_	*C*_3_	*C*_4_	*C*
0.0654	1.1221	0.5361	4.542	3.0

## Data Availability

Data are contained within the article.
